# Screening of the small molecule library of Meinox enables the identification of anticancer compounds in pathologically distinct cancers

**DOI:** 10.3906/biy-2104-14

**Published:** 2021-10-18

**Authors:** Aynura MAMMADOVA, Arif MERMER, Fatih KOCABAŞ

**Affiliations:** 1 Department of Genetics and Bioengineering, Faculty of Engineering, Yeditepe University, İstanbul Turkey; 2 University of Strasbourg CNRS France; 3 Biotechnology Department, Hamidiye Health Sciences Institute, Health Sciences University, İstanbul Turkey; 4 Meinox Pharma Technologies, İstanbul Turkey

**Keywords:** Screening, small molecules, Meinox small molecule library, cytotoxicity

## Abstract

Small molecules are widely used for the modulation of the molecular basis of diseases. This makes them the perfect tool for discovering and developing new therapeutics. In this work, we have established a library of small molecules in house and characterized its molecular and druglike properties. We have shown that most small molecules have molecular weights less than 450. They have pharmaceutically relevant cLogP, cLogS, and druglikeness value distributions. In addition, Meinox’s small molecule library contained small molecules with polar surface areas that are less than 60 square angstroms, suggesting their potent ability to cross the blood-brain barrier. Meinox’s small molecule library was also tested in vitro for pathologically distinct forms of cancer, including pancreatic adenocarcinoma PANC1, breast carcinoma MCF7, and lymphoblastic carcinoma RS4-11 cell lines. Analysis of this library at a dose of 1 μM allowed the discovery of potent, specific or broadly active anticancer compounds against pathologically distinct cancers. This study shows that in vitro analysis of different cancers or other phenotypic assays with Meinox small molecule library may generate novel and potent bioassay-specific compounds.

## 1. Introduction

Small molecule libraries accelerate the exploration of chemical leads that act as a starting point for novel therapeutics (Coussens et al., 2017; Bayram et al., 2018; Demirel et al., 2018; Beksac et al., 2020; Özenver and Efferth, 2020). With this approach, biologically and physiologically relevant assays can be rapidly tested, with the complex chemical space of thousands of small molecules. The discovery techniques for small molecules are commonly defined in phenotypic cell-based tests and biochemical target-based tests (Mohammadi et al., 2020). These include but not limited to target-based approaches, mechanism-modulated assays, phenotypic assays, combination assays, patient-derived cells for personalized therapeutic development, ex vivo tumor models such as organoids, spheroids, and tumor fragments (reviewed in (Coussens et al., 2017)).

A commonly used paradigm for the treatment of various human malignancies is combination drug therapy (Boztas et al., 2013; Bakhshaliyev et al., 2020). Additive and synergistic drug formulations in cancer care can turn weakly successful monotherapy treatments into regimens that deliver vigorous antitumor action (Daglioglu, 2017; Karpuz et al., 2018; Kacı et al., 2020). This can be explained in part by the interdependence of receptors that are important for the growth and survival of cancer (Gökbulut et al., 2015). However, because of the intricate molecular machinery that underlies tumor growth and progression, recognition of multiple interdependencies is difficult (Kaymaz et al., 2015). Further experiments are also required to eradicate these pathways of interdependence and drug resistance (Kara et al., 2021). This also requires development of new small molecule library that is suitable for high-throughput testing ( Zaka et al., 2018; Kanan et al., 2020). 

For the drug development, the exploitation of new chemical entities (NCEs) in various bioassays, and combinatorial studies, and to provide a novel, local and easy to access screening tool, Meinox has developed a novel library of small molecules. Meinox small molecule library includes over a thousand distinct and pharmacological molecules, and it is likely to be expanding to tens of thousands compounds soon with NCEs. 

In this work, a novel library of small molecules has been developed and its molecular and druglike properties have been characterized. Around a thousand small molecules from the Meinox repository were tested for their effect on cancer viability at a dosage of 1 μM for three different cancers. The goal of these compounds was to reduce the viability of MCF7 breast carcinoma, PANC1 pancreatic adenocarcinoma and RS4-11 lymphoblastic leukemia cell lines by at least 50%. For each cancer type, we have aimed to define specific hits with anticancer action. This study suggests that in vitro screening with the small molecule library of Meinox may generate novel and effective compounds related to the bioassays or cancer used.

## 2. Methods

### 2.1. Small molecule library preparations

The Meinox small molecule library chemicals are dissolved dimethyl sulfoxide (DMSO) as 10 mM main stocks. They have been diluted with PBS to obtain 100X working stocks before use in cell culture studies. List of compounds is provided on the http://www.meinoxtech.com/meinox-small-molecule-library.html. 

### 2.2. Computational analysis of Meinox library of small molecules

Meinox small molecule library includes over thousand druglike compounds in house, which is established with the support of medical chemists in Turkey, and antiparasitic/pandemic small molecule projects. Small molecules were characterized for their general molecular and lead/druglikeness properties. We analyzed their molecular weight, cLogP, cLogS, Polar surface area, fragment-based druglikeness value, and toxicity risk assessment for mutagenicity, tumorogenicity, irritating effects, and reproductive effects using DataWarrior (López-López et al., 2019). Histograms are prepared using online Shodor Histogram platform.

### 2.3. Cell culture and viability assays

Breast carcinoma cell line MCF7 (ATCC HTB-22), pancreatic adenocarcinoma cell line PANC1 (ATCC CRL-1469), and lymphoblastic leukemia cell line RS4-11 (ATCC CRL-1873) were cultured and passaged according to ATCC’s recommendations. Briefly, 10 × 10^3^ MCF7 and 7.5 × 10^3^ PANC cells were seeded into 96-well plates/well the day before small molecule treatments. A total of 200 × 10^3^ RS4-11 cells/well were passaged in the growth medium and treated with 1 μM of small molecules from Meinox library on the same day. DMSO (0.5%) treatments were used as a control. After four days of treatments, cancer viability was assessed by CellTiter (Promega) according to manufacturer’s protocol as we have done previously (Boztas et al., 2013; Turan et al., 2020).

### 2.4. Structural clustering and statistical analysis

Using Cactus server (https://cactus.nci.nih.gov/translate/), we have generated 3D SDF files of the ligands. Using ChemBioServer 2.0 (https://chembioserver.vi-seem.eu/index.php), hit clustering was performed. The distance form of Soergel (Tanimoto coefficient) and average linkage clustering were chosen. Statistical analysis was carried out by comparing chosen hits to the entire library of small molecules using the Student’s t-test. p values less than 0.05 were deemed significant.

## 3. Results

### 3.1. Analysis of molecular and lead/druglike properties of Meinox small molecule library

**Figure 1:**
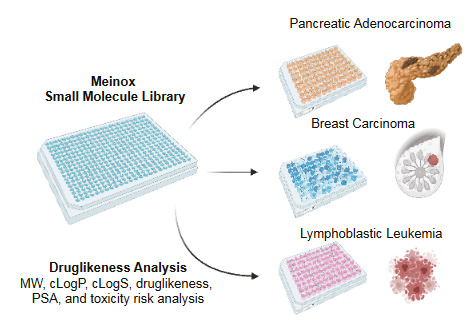
Meinox small molecule library was tested and validated in pancreatic adenocarcinoma (PANC1), breast carcinoma (MCF7), and lymphoblastic leukemia (RS4-11) in 1 μM final concentrations for cancer viability.

We established a library of small molecules with the support of medical chemists and molecular biologist who provided small molecules that are biologically relevant and subject to further characterization for drug discovery and development. First version of the Meinox small molecule library included 1000 small molecules. We sought to determine the molecular and druglike properties of small molecules by analyzing molecular weight, hydrophilicity (cLogP), aqueous solubility (cLogS), polar surface area (PSA), druglikeness value based on fragment analysis, and toxicity risk assessment for potential mutagenicity, tumorigenicity, irritating effects, and reproductive effects by computation tools. This is followed by in vitro analysis of compounds in breast carcinoma, pancreatic adenocarcinoma, and lymphoblastic leukemia cell lines (Figure 1).

**Figure 2:**
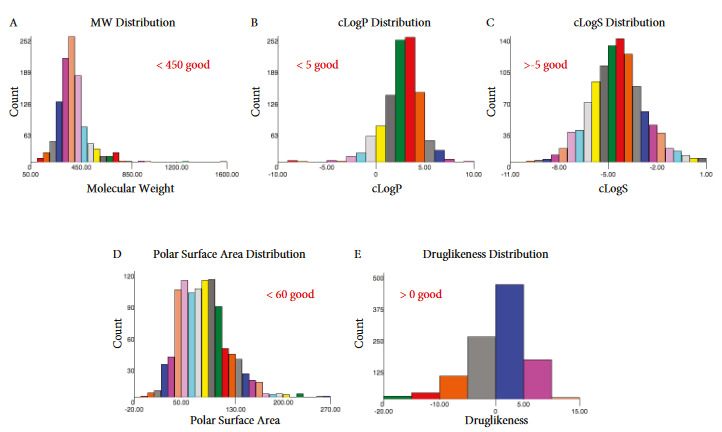
Analysis of molecular and lead/druglike properties of Meinox small molecule library. Distribution of A) molecular weight, B) cLogP (associated with hydrophilicity, absorption or permeation), C) cLogS (associated with aqueous solubility), D) polar surface area (associated with cell permeability and penetration into blood-brain barrier), and E) druglikeness.

Our analysis showed that majority (over 70%) of Meinox small molecule library compounds have molecular weight of 450 (Figure 2A), which is a good indication for druglikeness given that over 80% of traded pharmaceuticals have a molecular weight below 450. cLogP measurements provided potential hydrophilicity, absorption or permeation of studied compounds. We have found that majority of Meinox small molecule library demonstrated cLogP values smaller than 5.0, similar to pool of traded small molecule drugs in the market (Figure 2B). In addition, aqueous solubility of compounds were in the range of over –4 for cLogS values showing that majority of compounds are likely to have some degree of aqueous solubility (Figure 2C). Moreover, we determined the distribution of compounds based on their potent cell permeability and ability to penetrate the blood-brain barrier by analysis of polar surface area. Given PSA values in the drugs that are likely to act on the central nervous system have below 60 square angstroms, we considered compounds in the Meinox library to likely to have ability act in this difficult to targeted region of the body. We have found that there is a large number of compounds that fits into PSA criteria (Figure 2D). Furthermore, druglikeness based on the assessment of fragment-based approach in Meinox small molecule library showed that about 70% of the compounds have a positive druglikeness value (i.e. >0), which is in correlation with known drugs (Figure 2E). 

### 3.2. Meinox small molecule library allows identification of compounds specifically could block breast, pancreas and leukemia growth

Meinox small molecule library compounds are maintained in DMSO as 10 mM stocks and diluted to 100X working stocks before use in cell culture experiments. Up to 963 compounds were tested in vitro in this study. The PANC1 pancreatic adenocarcinoma cell line, MCF7 breast carcinoma cell line, and the RS4-11 lymphoblastic leukemia cell line were used to determine potent anticancer compound in the Meinox small molecule library. Cancer cells were seeded into 96-well plates and treated with 1 μM of small molecules from the Meinox library. Then, cancer viability was assessed after four days of treatments. 

**Figure 3:**
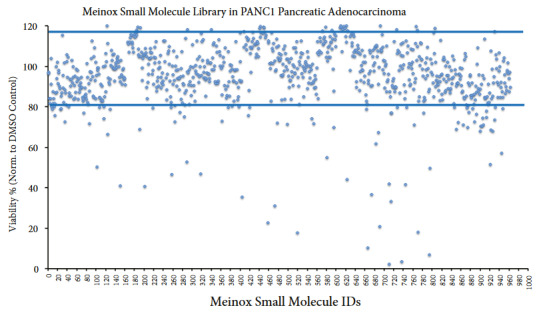
Analysis of cell viability posttreatment of PANC1 pancreatic adenocarcinoma cell line with the Meinox small molecule library.

Treatment of Meinox small molecules with PANC1 cells at 1 μM final dose revealed twenty-one compounds that decrease cell viability at least 50% (Figures 3 and S1). With the PANC1 cell viability assay, we obtained an overall viability of 97.2 ± 17.2% with Meinox small molecules. Mx00300, Mx00710, Mx00737, and Mx00794 demonstrated the most robust cancer cell viability reduction in PANC1 pancreatic adenocarcinoma cell line. 

**Figure 4:**
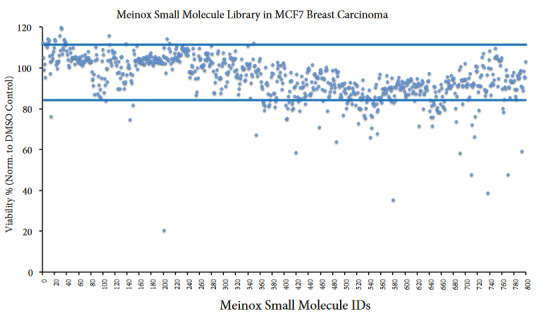
Analysis of cell viability posttreatment of MCF7 breast carcinoma cell line with the Meinox small molecule library.

Treatment of MCF7 cells with Meinox small molecules (800 compounds) at 1 μM final dose showed five compounds that decrease cell viability more than 50% (Figures 4 and S2). We achieved an overall average of 95.0 ± 7.3% viability with MCF7 cells and Meinox small molecule treatments. Treatment with Mx00201 at 1 μM final dose resulted in about 80% reduction in cell viability in MCF7 breast carcinoma cell line. 

**Figure 5:**
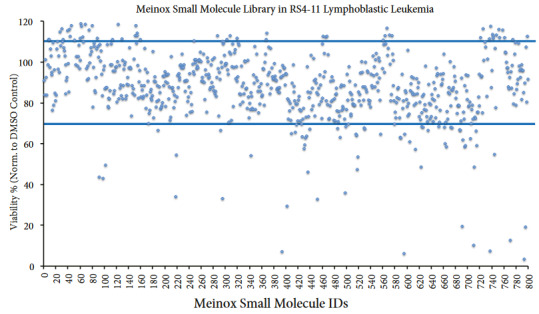
Analysis of cell viability posttreatment of in RS4-11 lymphoblastic leukemia cell line with the Meinox small molecule library.

The treatment of Meinox small molecules (800 compounds) in RS4-11 cells at 1 μM final dose showed twenty compounds that minimized cell viability by at least 50% (Figures 5 and S3). With the RS4-11 cell viability assay, we achieved an average viability of 89.5 ± 17.9% with the small molecules of Meinox. Mx00394, Mx00595, Mx00737 and Mx00794 showed the most robust decline in the viability of RS4-11 pancreatic adenocarcinoma cells.

### 3.3. Analysis of common anticancer compounds from Meinox small molecule library demonstrates potential druglike compounds

**Figure 6:**
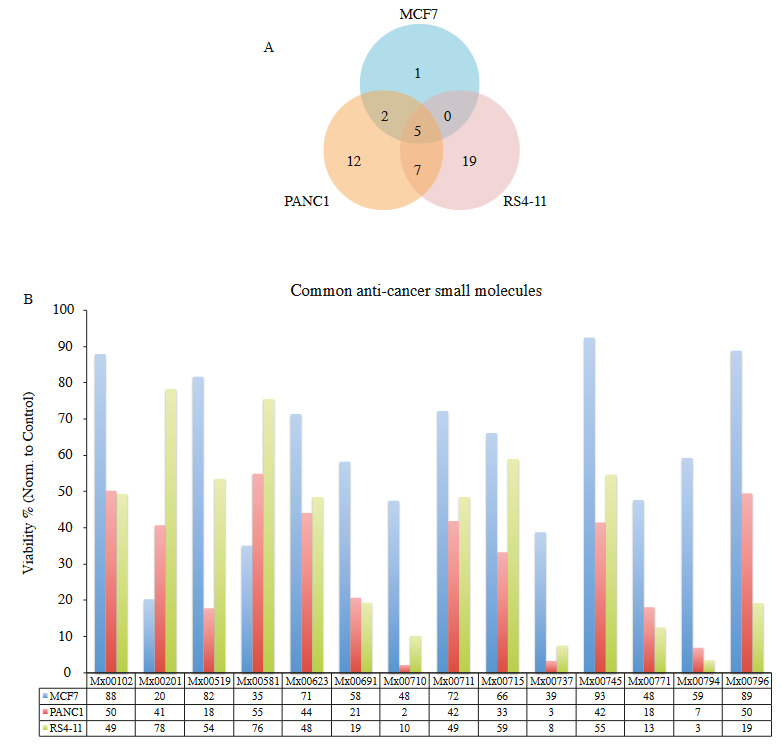
Analysis of small molecules in cell viability in the Meinox small molecule library. A) Distribution of common and cancer type specific small molecules identified with at least 40% reduction in cell viability in MCF7, PANC1 or RS4-11 cancers. B) Percent viabilities of common thirteen small molecules.

**Figure 7:**
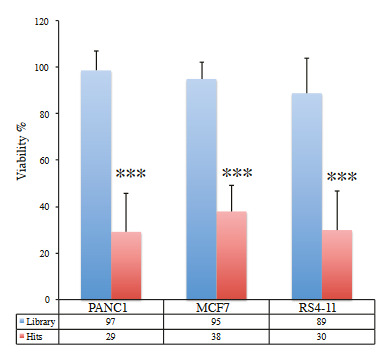
Analysis of average cell viability of whole library and common hits. We have calculated cell viability of whole small molecule library treatments and compared to selected hits for each cancer. *** indicates at least p <10–16.

We wondered if the identified anticancer hits are common or unique against three distinct cancer types studies (Figures 6A and 6B). We have found that only five compounds had common and broad anticancer activity against breast, pancreas and blood cancer cell lines studied. These compounds included Mx00691, Mx00710, Mx00737, Mx00771 and Mx00794**. **Intriguingly, pancreatic adenocarcinoma PANC1 and lymphoblastic leukemia RS4-11 cell lines shared seven additional common anticancer compounds effective at 1 μM dose. MCF7 and PANC1 shared two compounds (Mx00201 and Mx00581) with anticancer properties that do not affect RS4-11 cell viability at 1 μM dose. Mx00420 compound only reduced MCF7 cell viability at 1 μM dose, but not PANC1 or RS4-11 cell lines. Mx00300, Mx00666 and Mx 00458 were anticancer hits that were only observed in PANC1 cell viability assays. Besides, RS4-11 had over 19 unique hits from Meinox small molecule library that lowered cell viability at least 50%. Furthermore, when we compared average cell viability of whole small molecule library with selected hits in each cancer types, we have found significant difference (p < 10^–16^ and smaller) (Figure 7).

**Figure 8:**
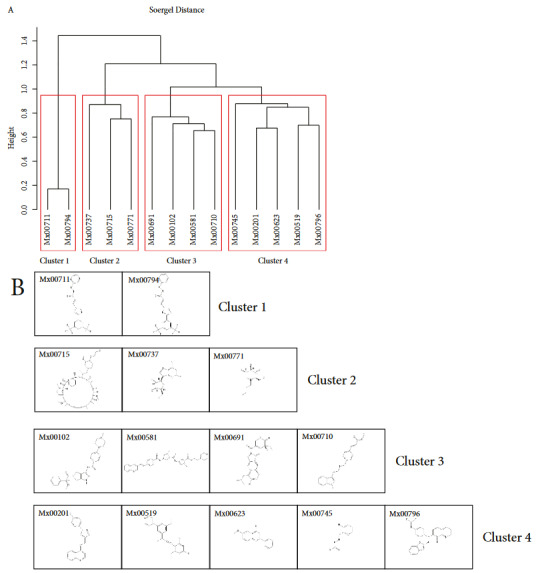
Structural analysis of hits. A) Structural classification of common anticancer hits. B) Structures of identified anticancer compounds from Meinox library of small molecules. Mx = Meinox compound ID.

Common hits identified from this study demonstrated structural similarities between compounds (Figure 8A). Analysis of common 14 hits (double or triple hits) by Tanimoto clustering for MCF7, PANC1 or RS4-11 showed that they could be classified into four groups (Figure 8B). In addition, we determined druglikeness properties (Table). We found that 10 of 13 hits had below 450 MW. A total of 11 of 13 hits demonstrated cLogP < 5. A total of 7 of 13 hits had cLogS value bigger than –4. A total of 2 hits had PSA of below 60. A total of 7 of 13 hits had druglikeness value >0 indicating their potent drug potential. Furthermore, we computationally determined potent toxicity risks namely mutagenicity, tumorigenicity, irritant or reproductive effects of hits. Majority of hits had no almost none toxicity risks.

In short, we have established a library of small molecules with potentially and biologically active new chemical entities. Analysis of this library at 1 μM dose allowed discovery of potent, unique or broadly active anticancer compounds against pathologically distinct cancers. Analysis of some hits displayed enhanced properties of solubility and permeability and unusual structures that could be further studied as potent lead compounds. This research also indicates that in vitro analysis of different cancers or resistant disease models or other phenotypic assays with Meinox small molecule library could yield and recognize novel potent compounds unique to the utilized bioassay.

**Table:** Druglikeness analysis of identified common anticancer compounds. Identified small molecules from Meinox library of compounds were analyzed for their MW, cLogP, cLogS, Polar surface area, druglikeness, and predicted for mutagenicity, tumorigenicity, reproductive effect, and irritation.

Meinox ID	Total MW*	cLogP**	cLogS***	Polar surface area****	Druglikeness*****	Mutagenicityprediction	Tumorigenicprediction	Reproductiveeffect prediction	Irritantprediction
Mx00102	474.6	0.7341	–2.603	93.8	10.497	None	None	None	None
Mx00201	336.8	2.7981	–4.147	52.83	–3.4385	None	None	None	None
Mx00519	373.5	3.7552	–6.194	99.94	–4.9099	High	Low	High	None
Mx00581	631.7	3.4038	–4.399	122.52	6.0525	None	None	None	None
Mx00623	251.3	3.3101	–3.822	42.35	–1.5828	None	None	None	None
Mx00691	421.5	0.461	–1.959	103.2	6.6593	None	None	None	None
Mx00710	349.4	2.6014	–4.478	77.15	1.0293	None	None	None	None
Mx00711	443.3	2.572	–4.583	97.62	–4.5155	None	None	Low	None
Mx00715	958.2	6.4181	–6.634	204.66	1.9808	None	None	None	None
Mx00737	285.2	–1.2693	–2.91	139.54	–2.2482	None	None	None	None
Mx00745	195.2	–0.0922	–2.203	121.41	2.2769	Low	None	None	None
Mx00771	219.3	–0.8458	–0.277	84.16	2.5386	None	None	None	None
Mx00794	442.3	3.5729	–5.378	84.73	–4.5155	None	None	Low	None
Mx00796	449.5	5.3826	–6.258	95.51	–2.2098	None	High	None	None

*Good < 450, **Good < 5, ***Good > –4, ****Good < 60, ****Good > 0).

## 4. Discussion

Pharmaceutical modulation of small molecule diseases follows a variety of common features that allow us to foresee when a novel chemical agent with potent biological activity could become a medicine in the future. One of the underlying general characteristics of traded drugs is their small molecular weight, but the production of higher activity compounds is often correlated with elevated molecular weights following the discovery of hits. Compounds of larger weights, however, are less likely to be absorbed and thus enter the area of influence. Thus, the wish of a drug developer is to strive to keep molecular weights as small as possible. Thus, the novel screening library should follow these features as envisaged and promote the further production of the drugs from the onset of the identified hit compounds. To this end, Meinox small molecule library contains 818 compounds (81.8%) with a molecular weight less than 450.

A well-known measure of the hydrophilicity of the compound is the logP value of a compound, which is the logarithm of its partition coefficient between n-octanol and water log. The weak absorption or permeation is caused by low hydrophilicities and thus high logP values. Compounds have been seen to have a fair tendency to be well absorbed; ones logP value must not be greater than 5.0. To this end, the small molecule library of Meinox established in this study has been examined for cLogP values found to have 926 compounds (90.26%) of cLogP values less than 5.

Aqueous solubility affects the absorption and distribution properties of a compound greatly. A low solubility is usually followed by slow absorption, meaning that poorly soluble compounds are avoided generally in drug discovery and development platforms. The logS value estimation is based on mol/litre calculated solubility at base 10 of the logarithm. More than 80% of medications on the market have a logS value higher than –4. In comparison, Meinox small molecule library established in this study contains 375 compounds (37.5%) with a value greater than –4 and 641 compounds (64.1%) with a value greater than –5 cLogS.

The polar surface area (PSA) is known as the surface amount of all polar atoms (oxygen, nitrogen, sulfur and phosphorus), including hydrogens that are also attached. PSA is a widely used metric in medicinal chemistry for cell permeability optimization. It is commonly thought that molecules with a polar surface region of more than 140 square angstroms are bad at permeating cell membranes. In addition, for molecules to cross the blood-brain barrier and act on central nervous system receptors, PSA should be less than 60 square angstroms. It seems that newly established Meinox small molecule library in this study includes 927 (92.7%) compounds with PSA values below 140, and 293 (29.3%) compounds with PSA values below 60. 

Earlier estimates of possible toxicity may minimize errors in the later stages of drug development due to cytotoxicity, tumorigenicity, or reproductive issues. The computational prediction methods are based on a preset structural fragment that triggers toxicity warnings if located in the current structure. Risk warnings by computational analysis are by no means supposed to be a predictor of toxicity that is entirely accurate. In the lack of risk signs, it could not be inferred that a given product will be absolutely free of any harmful effects. The prediction process depends on the structural fragment which gives rise to toxicity warnings should they be located in the RTECS database. We ran a number of toxic compounds and a set of possibly nontoxic compounds through the forecast to test the efficiency of the toxicity prediction in the newly established Meinox small molecule library in this study. The frequency of occurrence of any fragment (core and fabricated fragments) within all compounds of that toxicity class such as mutagenicity, tumorigenicity, reproductive or irritant effect was calculated by the substructure search process. Based on the premise that the drugs sold are essentially free from toxic effects, any fragment was deemed a risk factor if it always existed as a substructure of hazardous substances.

Meinox small molecule library allowed us to identified both new (i.e. Mx00921, Mx00945) and previously reported anticancer compounds (i.e. Mx00691, Mx00710, Mx00737 and others). Intriguingly, several of the identified common anticancer hits which reduced viability of three cancers, pancreas, breast and blood cancer cell lines, had previously reported or recently been approved as antineoplastic agents through drug repurposing studies. Mx00691, also known as Topotecan, has been used in the treatment of colorectal, ovarian and nonsmall cell lung cancer as antineoplastic agents (Noronha et al., 2020). It is a derivative of camptothecin which binds to the topoisomerase I-DNA complex. Mx00710, also known as Panobinostat, is an antineoplastic and inhibitor of histone deacetylase agent used with other agents of refractory or relapsed myeloma (Mamdani and Jalal, 2020). It may also contribute to G2/M phase cell cycle blockage and apoptosis. Mx00737, also known as Fludarabine, is an antimetabolite analog of vidarabine that poses antineoplasty (Barreca et al., 2020). Mx00771, also known as Miglustat, is analogue of D-glucose and used in the therapy of type 1 Gaucher disease (Stirnemann et al., 2017). Intriguingly, to delay the development of multiple myeloma, researchers have recently repurposed the licensed drug miglustat (Ersek et al., 2015). Mx00794, also known as (Gravina et al., 2014), is a new agent that is being tested in a number of viral indications as well as in inflammatory disorders as an oral selective inhibitor of nuclear export (SINE). Additionally, the prevention of canine tumors including lymphoma is being investigated (Sadowski et al., 2018). 

Different carcinomas might have different dependencies of different molecular pathways. Therefore, while some anticancer compounds are blocking the growth of a particular cancer, it may not affect another type of cancer cell line or carcinoma. This type of anticancer compounds could be key in developing pathway/cancer specific precision medicine approaches. Besides, some anticancer compounds have broad activities; they block cancer cell growth almost in every type of cancer that have been treated. These compounds are usually cytotoxic to cells, thus they may show cytotoxicity to healthy cells as well. This limits use of these anticancer compounds in future therapeutics if benefit to harm ratio is not good enough. 

In brief, we have developed a library of small molecules with theoretical and biologically active chemical entities. The potent anticancer compounds were determined for the PANC1, MCF7, and RS4-11 cell lines. The 1 μM dose evaluation of the library revealed strong, unique or highly active anticancer compounds for pathologically distinct cancers. Hit analysis reveals higher solubility and permeability and unique structures which can be further analyzed as effective lead compounds. This research also shows that in vitro analysis of different cancers or disease-resistant models or other phenotypic assays with the Meinox small molecule library could yield and define novel potent compounds unique to the bioassay used.
